# Supplementary material for: Gender differences in perceptions of “joint” decision-making about spending money among couples in rural Tanzania
Source: PLoS One. 2024 Jun 12;19(6):e0302071. doi: 10.1371/journal.pone.0302071 (PMC11168642; doi:10.1371/journal.pone.0302071)
Supplement: Attachment — Submitted filename: PONE-D-23-01170_reviewer_comments.docx [file pone.0302071.s001.docx]

Overall: this manuscript incorporates both a quantitative and qualitative exploration of household decision-making as part of a larger project in Tanzania. A comparison of spouses’ responses led to a robust discussion of the complexities of decision-making and the development of a spectrum that can be helpful in understanding household decision-making. Overall, this is an important topic and one that those working in gender are continuing to struggle with. At the same time, there are ways that the manuscript can be strengthened – from the framing in the introduction to specifics in the methods and results, to better contextualization of results and interpretation in the discussion – that would help to strengthen the manuscript overall. Please see more detailed comments below.

Abstract:

In the beginning part of the abstract as well as at the end, I wonder if it is more compelling to explain why, for health and wellbeing, it is important to explore dynamics around decision-making.

The second sentence of the abstract is run-on and it needs to be separated/revised.

Suggest removing “very” from sentences as it is a vague descriptor.

Based on what is written in the abstract, it is unclear how the complexities in decision-making led to the conclusion that couples/households are on a spectrum from no cooperation to full cooperation.

Introduction:

Paragraph 1: Can you expand/elaborate, give specifics on what conflict and cooperation about in households can look like?

Paragraph 1, line 4-5: Positive behaviors such as?

What development objectives does ignoring gender dynamics hinder?

Is the issue defining gender equality, or implementing and putting into practice and contextualizing what gender equality looks like?

Paragraph 2: women’s empowerment is related to gender equality, but distinct. I think the relationship between these concepts could be clarified. In addition, a lot of different concepts are covered in this paragraph, and how they relate, in particular how/why decision-making is important for improving gender equality, should be expanded.

I think the organization of the introduction could be improved to strengthen and improve clarity. I think it might be stronger to start the introduction making the important linkage between decision-making and health outcomes, which currently comes in paragraph 3, and why we should understand decision-making and then introduce key concepts. That might help the flow as the introduction currently goes back and forth between gender equality and decision-making. It might also be important to discussion the distinction between women’s decision-making and joint decision-making a bit earlier before page 4.

Page 5: “Research have found” – in what contexts? In addition, a lot of studies are summarized in this paragraph, and it might help to add a topic sentence or concluding sentence that summarizes/synthesizes the overarching key point in how these studies vary.

It might be helpful to, in the last paragraph of the introduction, contextualize the aims and clarify the location of the study.

Methods:

In the first paragraph, when contextualizing the larger project, it might be helpful to describe briefly the way that Phase A/B results were used as part of the project – were they formative, evaluative, etc.? Phase B focused on household-level attitudes and practices related to what exactly? What nutrition and health behaviors in particular?

It would be helpful to introduce the factors shown to be associated with decision-making in previous research earlier in the introduction if they were considered as part of sampling (e.g., age, education, etc.).

There is a lot of detail included about the overall study, but I wonder if detail about data collection that is not relevant to the current manuscript should be removed to focus specifically on the methods that led to the data analyzed here.

Data analysis approaches for quantitative data should be included in the data analysis section as well. How was concordance assessed?

In addition to the analysis of the qualitative transcripts, were particular approaches used to analyze the pile sort activity data? I know space considerations are important to consider, but the results seem to be important to include.

Results:

Suggest being specific in the presentation of results rater than saying “about.” For example, not “about 8 years” but presenting the specific number.

Any reflections on men’s reflections on DM with one wife vs. another?

p. 10, 2^nd^ paragraph of results: this reads more like interpretation and should be better placed in the discussion.

Given the small sample size, I think presenting statistics with 1 decimal point seems more appropriate. This would make Table 1 consistent with Table 2.

The summary of concordance/discordance in Table 3 (and Table 4) could be elaborated in the narrative. Furthermore, it is important to look not only at whether they were concordant or not, but also what that concordant response was. Can that be broken out/disaggregated further? For example, were they concordance that men made the decision, that women mad ethe decision, etc.?

In describing the example households, it might be helpful to provide more background details about the household (perhaps from the demographic characteristics?). I would also perhaps develop a name for the households rather than using labels like “household 73.” In the tables with quotes from participants, the title of the tables seems to be the nickname/label that could be used. However, it’s important to make this consistent with the sub-heading title where you describe that/those households. Currently, they are slightly differently described.

While the tables with illustrative quotes are helpful, is there space to incorporate a few quotes into the main body of the results section?

Discussion:

One question that has come to mind in this analysis is that the framing is around household decision-making, but the analyses seem often focused on the husband and wife. Is there space to elaborate on the role of other family members in household decisions?

The first two paragraphs in the discussion could perhaps be synthesized with what is in the introduction so that the discussion dives right into the key findings and situating them within the context of existing literature and identifying programmatic implications.

Page 27, last paragraph: remove contraction in first sentence.

It would be helpful to see more concrete recommendations related to future research and how those findings can be used to inform programs, particularly in light of how the paper has been framed.

Can more attention be given to the discussion of limitations in the discussion section? For example, a discussion of transferability and context might be important to include.

Conclusion: I would suggest being careful saying “significant differences” when not referring to a statistical association.
